# Seismic performance of a new precast concrete frame joint with a built-in disc spring

**DOI:** 10.1038/s41598-023-32447-1

**Published:** 2023-04-01

**Authors:** Qi Chen, Yongjun Qin, Yi Xie, Chen Yang

**Affiliations:** College of Civil Engineering and Architecture, Xinjiang University, Urumqi, 830063 China

**Keywords:** Structural materials, Civil engineering

## Abstract

A new, precast concrete frame beam-column connection is designed in this research. The connection adopts the assembly mode of the precast column and seam area jointly to maintain the integrity of the joint area and increase the assembly efficiency. Based on the conventional grouting sleeve connection, a disc spring device is constructed on the beam end to improve the ductility of the joint. Ten connecting specimens were tested under low cyclic loads, including two monolithic connections, four ordinary precast connections, and four new precast connections. The test parameters included the joint type and axial pressure ratio, and the difference in the seismic performance was determined by evaluating the failure mode, hysteresis characteristics, stiffness degradation, energy dissipation, and shear deformation of the joint area. Compared to monolithic connections, conventional precast connections display similar hysteresis characteristics. Although their ductility is slightly lower, their bearing capacity is higher. Compared with the previous two connections, the new connection with the built-in disc spring device has superior seismic performance. The axial pressure ratio is a significant aspect in determining the failure mode of the precast connection, and the specimen exhibits less shear damage at a larger axial pressure ratio.

## Introduction

Precast concrete (PC) frame structures have the advantages of enhancing construction quality, enhancing construction efficiency, conserving labor, saving energy, and reducing emissions; consequently, the strategic topic of new building industrialization based on PC frame structures has received increasing attention in recent decades^1–4^. However, the poor seismic performance of PC frame structures during earthquakes has been the reason limiting the widespread use of PC frame structures in high-intensity areas^5^. It is well known that the seismic performance of PC frame structures is highly correlated with the reliability of the PC beam-column connection. In many experimental studies, it has been found that the phenomenon of PC building collapses that are caused by the failures of PC beam-column connections is the most common^6,7^. Consequently, evaluating the seismic performance of PC frame beam-column connections is a prerequisite for the widespread implementation of prefabricated concrete frame structures in high-intensity locations.

Ductility and energy consumption have been extensively investigated as two crucial aspects influencing the seismic performance of PC frame connections. The assembly mode of precast components has a direct effect on the energy consumption of the PC frame structure, and several researchers have improved the energy efficiency of the PC connection by inventing various assembly modes. Currently, the most prevalent mode of assembly is to precast beam and column members separately, which are subsequently brought to the site for assembly and poured with concrete at the seam area^8,9^. Different forms of assembly present distinct issues. After pouring concrete into the column^10,11^, the pouring area of the column is too large, resulting in inefficient construction, and the emergence of weak points in the column is detrimental to its energy consumption. Pouring concrete at the end of the beam^12^ can ensure the integrity of the column and conform to the design principle of a “strong column and weak beam” in seismic design; however, the longitudinal ribs in the beam cannot be continuous at the seams, and it is difficult to ensure effective stress transmission during earthquakes. Together, precast components and seams ensure the integrity of the node region and cause the joint area to have a better energy consumption performance^5,13^. The reliable form of the reinforcement connection inside the precast member is another key factor affecting the energy consumption capacity of the PC frame connection, and the common lap connection requires a long lap length and poor bond strength^14,15^. Studies that improve lap connections have demonstrated that while they can improve their seismic performance, the complex fabrication and construction process makes it difficult to promote in precast components^16,17^. Sleeve connections are extensively utilized for their simple operation, reliable joints, and excellent stress transfer capabilities; however, the ductility of PC components is weak due to their inherent features^5,18^.

Attaching energy consuming devices in the joint area or installing energy consumption dampers outside or inside the joint can increase the ductility and energy consumption of the precast connection, according to related studies. Ertas et al.^11^ designed a ductile PC framework connection and compared the energy dissipation of cast-in-place, welded composite, and bolted connections, concluding that the enhanced bolted connection may be suitable for usage in seismically active regions. Morgen and Kurama^19^ use a tribe-damper-assisted connection design to improve the energy consumption, and the analytical findings indicate that the design of the tribe-damping precast frame energy dissipation levels. Vidjeapriya and Jaya^20^ mounted triangular stiffening ribs as energy-consuming pieces at the junction of beams and columns of precast specimens, which demonstrated satisfactory performance in terms of the energy consumption and ductility when compared to monolithic specimens. Huang et al.^21,22^ proposed a new self-centered precast concrete beam-column connection with a variable friction damper (VFD), and the experimental results showed that this connection method can achieve significant and reliable energy consumption levels while maintaining the self-centering ability. Luci et al.^23^ devised a replaceable energy-consuming connector (REDC) that provides stable hysteresis performance and high low-cycle fatigue performance under cyclic reverse loading conditions. In most studies regarding additional energy-consuming parts, it was found that the joint construction of the energy-consuming parts was inconvenient or difficult to replace and repair. Moreover, the connection that sets the external damper has a greater energy consumption capacity. However, the space occupation affects the use, and the cost is higher; relatively simple, practical, and reliable internal dampers have been fully developed in the study of PC framework connections.

Disc springs manufactured from a high-strength alloy have simple production, good stiffness, a high-pressure capacity, and outstanding mechanical properties. Different combinations of superpositions and cross-fittings can achieve different stiffness and deformation capabilities, and because they can provide a certain degree of damping and dissipation of seismic energy through cone and edge friction, they are gradually being implemented in the field of construction and engineering industries. In recent years, some new energy-consuming supports with pre-pressurized disc springs have been developed. The results of the cycle test demonstrate that the new energy-consuming supports have reliable energy dissipation capabilities, and there is almost no damage after the test, allowing them to be reused. The energy-consuming building structure supported by the assembly disc spring has a significant reduction in the displacement peaks and residual deformations^24–27^. Currently, DSD is used in the corner of shear walls due to its stable recovery performance, which allows the shear walls to center themselves. Xiao et al.^28,29^ created a shear wall (SC-SW) utilizing a disc spring device, and the experimental results showed that the bearing capacity of SC-SW was lower than that of conventional shear walls, while shear walls incorporating DSD had a superior deformation ability and energy consumption. Xu et al.^30^ enhanced the wall corner disc spring device, and the numerical simulation and test results showed that the bearing capacity of this RC wall was higher and the energy consumption capacity was improved. Based on this, Xu et al.^31^ designed a tensile-pressure coupling DSD that enhanced the SC-SW bearing capacity and initial stiffness. However, the use of DSD in PC framework connections has not been documented; however, the superior deformation capability and energy consumption capability of the disc spring will inevitably provide a new idea for the design of PC frame connections.

**Figure 1:**
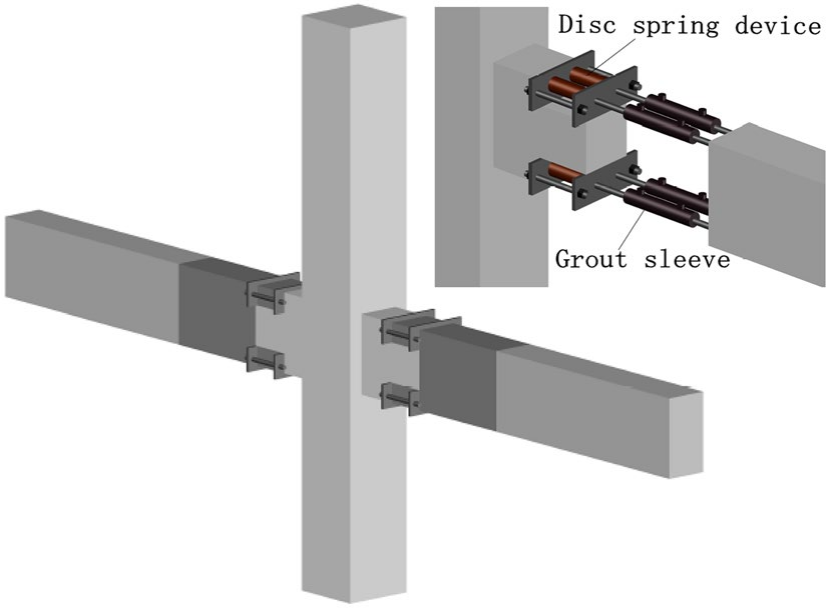
Schematic diagram of the joint of the new PC framework.

In summary, a new PC frame structure system is proposed in this paper, and conventional PC frame connections as well as a new form of PC frame connections with a built-in disc spring device (refer to Fig. 1) are developed. The connection adopts the precast assembly mode of the column and seam together, which ensures the integrity of the joint area and conforms to the design principle of the “strong column and weak beam” design idea. The prefabricated concrete frame structure based on grout sleeve connections has an excellent seismic performance. The longitudinal ribs in the precast beam are connected using grout sleeves, and the disc spring device is built into the beam end. The seismic performance test evaluates the strength, stiffness, ductility, energy consumption characteristics, and joint area shear deformation capacity. The effects of the joint type, assembly scheme, axial pressure ratio, and other parameters on the seismic performance of PC frame connections are examined.

## Experimental program

### Design and details of the specimens

**Figure 2:**
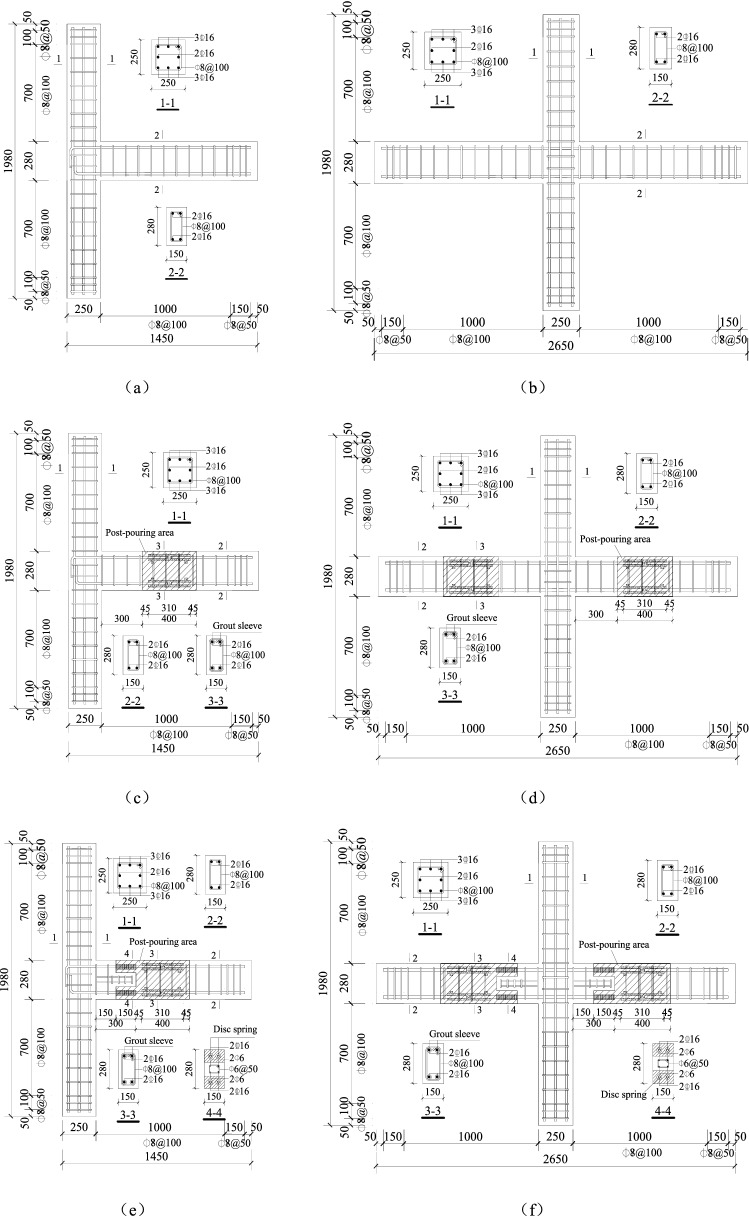
Configurations and reinforcement details of the test specimens (unit: mm) (**a**) ERC2; (**b**) IRC2; (**c**) EPC2 and EPC4; (**d**) IPC2 and IPC4; (**e**) EPCD2 and EPCD4; (**f**) IPCD2 and IPCD4.

To investigate the seismic resistance of the new precast concrete junction proposed in this test, two monolithic specimens (ERC2 and IRC2), four regular precast specimens (EPC2, EPC4, EPCD2, EPCD4), and four new precast specimens (IPC2, IPC4, IPCD2, and IPCCD4) were fabricated. Details of the ten specimens are summarized in Table 1. The exterior and interior connections select the 1/2 shrink model of the beam-column connection in the 6-layer reinforced concrete frame structure, the prototype structure seismic fortification intensity is 8 degrees, the ground peak acceleration PGA is 0.2 g, the frame structure standard layer height is 3.9 m, the bottom layer height is 4.2 m, the longitudinal span is 4.8 m, the transverse span is 4.2 m, the column section size is 500 mm × 500 mm, the beam section size is 300 mm × 550 mm, the column section size is 5.5 kN/m^2^, and the live load is 2.0 kN/m^2^. To avoid shear failure in the core region of the connection, as well as the bending failure of plastic hinges on the column, the connection should have sufficient strength to prevent shear failure of the core region of the connection before the failure of the beam and column member occurs. All beam-column connections are designed in accordance with the principles of the “strong column, weak beam”, “strong shear, weak bend”, and “strong joint, weak component” concepts in the “Code for Seismic Design of Buildings” (GB50011-2010)^32^, with a shear force increase coefficient of 1.5 and a bending moment ratio of 1.7. The reinforcement rate of the connection is identical to that of the prototype, which satisfies the minimal reinforcement rate. The performance of the proposed novel precast components is anticipated to be superior to that of the monolithic specimens if the aforementioned design approach is utilized. Figure 2 illustrates the shape, size, and reinforcement details of the test specimens.

**Table 1:** Summary of the specimen information.

Specimen	Type	Axial compressive ratio	Assembly schemes
ERC2	Exterior, monolithic	0.2	–
EPC2	Exterior, precast	0.2	Grout sleeve
EPC4	Exterior, precast	0.4	Grout sleeve
EPCD2	Exterior, precast	0.2	Disc spring; grout sleeve
EPCD4	Exterior, precast	0.4	Disc spring; grout sleeve
IRC2	Interior, monolithic	0.2	–
IPC2	Interior, precast	0.2	Grout sleeve
IPC4	Interior, precast	0.4	Grout sleeve
IPCD2	Interior, precast	0.2	Disc spring; grout sleeve
IPCD4	Interior, precast	0.4	Disc spring; grout sleeve

### Connection description

**Figure 3:**
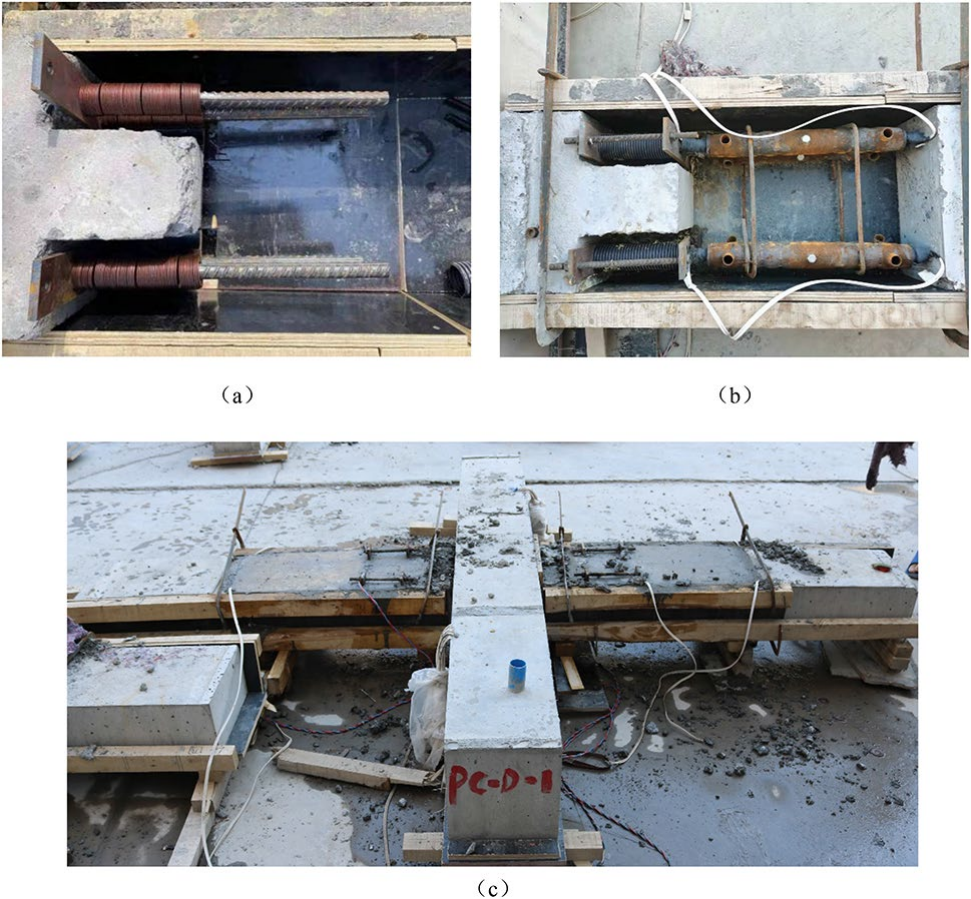
Assembly process of the specimen.

Monolithic specimens and precast components are manufactured in the factory and then transported to the laboratory after the strength of the reinforced concrete components meets the requirements. Additionally, the precast components are assembled in the laboratory. A detailed drawing of the assembly process is shown in Fig. 3. The following are the technical aspects of the assembly procedure.

After 28 days of maintenance, the precast components are assembled, and the surface of the precast concrete is roughened to strengthen the bonding strength of the new and old concrete surfaces and ensure the integrity of the precast specimens. The precast column is placed horizontally. (1) The disc spring is attached to the longitudinal bar at the beam end of the prefabricated column, reserving screw holes at the upper and lower ends of the left and right steel splints for its attachment to fix the disc spring. To avoid premature concrete crushing around the disc spring device, plastic film was wrapped around the disc spring device (Fig. 3a,b). (2) A full grouting sleeve is inserted on the beam end longitudinal rib of the precast column, the precast beam is moved, the reserved longitudinal rib is inserted into the grouting sleeve, the position of the precast beam is adjusted, the 400 mm post-pouring belt is reserved, and a laser level is used to verify the position of the precast beam and column (Fig. 3b). Regarding the regular precast connections, Step (a) is omitted. The grout sleeve is grouted with high-performance grout from the grouting port, and when the grout gushes out of the exhaust port, it is considered to be completed. The pouring belt is carried out after the grouting material reaches a certain strength, and the maintenance continues after the pouring is complete (Fig. 3c).

### Material properties

**Figure 4:**
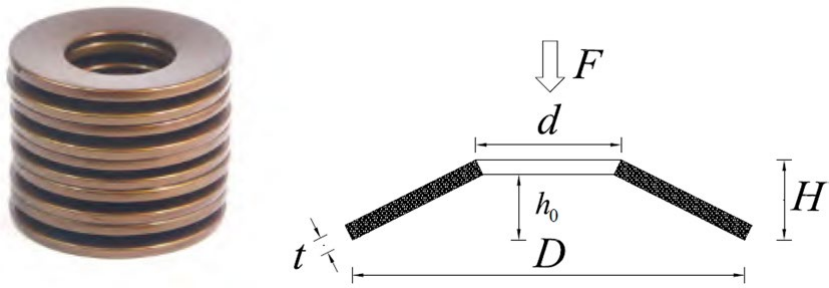
Disc spring and its geometric parameter.

**Figure 5:**
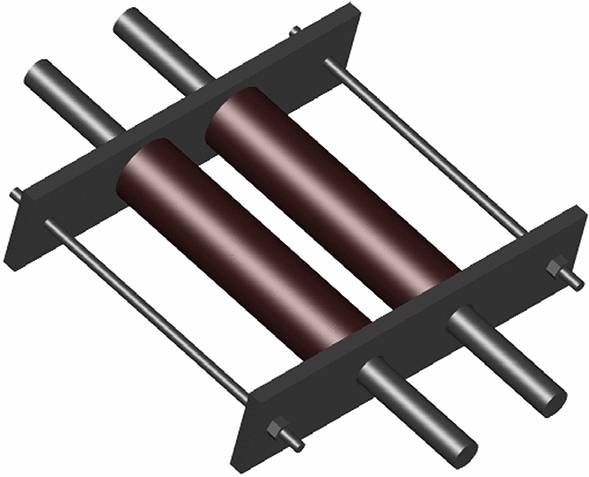
Configuration of the disc spring device.

Ready mixed C40 concrete is used for the monolithic specimens and precast components, and C50 fine aggregate concrete is used for the post-pouring area. By the “Standards for the test methods for the mechanical properties of ordinary concrete” (GB/T 50081-2016)^33^, six 150 mm × 150 mm × 150 mm pieces are poured alongside the precast components and after the connecting pieces are poured. After 28 days of curing under the same conditions as the test specimen, the cube compressive strength and other parameters are measured by the electrohydraulic pressure tester (refer to Table 2). The high-performance grouting materials utilized in the GTJQ grouting sleeve are listed in Table 3. Regarding the longitudinal bars of all connections, HRB400 steel bars with a diameter of 16 mm and a specified yield strength of 400 MPa are utilized. HPB300 steel bars with a diameter of 8 mm are utilized for the stirrups of the longitudinal bars. The specimens of each type of steel bar are subjected to tensile tests in accordance with “Metallic materials-Tensile testing” (GB/T 228.1-2010)^34^. Table 4 is a summary of the mechanical properties of the reinforcement. The disc spring used in the new connections is an ordinary series disc spring, with the specifications of D35.5 × 18.3 × 2 × 2.8 × 0.8. The relevant parameters and mechanical properties of a single disc spring are obtained from “Disc spring” (GB/T 1972-2005)^35^, as shown in Fig. 4 and Table 5. Figure 5 depicts a structural diagram of the disc spring device installed within the new connection for this test.

**Table 2:** Mechanical properties of the concrete.

Concrete grade	*f*_*cu*_ (MPa)	*f*_*c*_^'^(MPa)	*f*_*t*_ (MPa)	*E*_*c*_ (GPa)
C40	52.6	35.2	2.6	30.2
C50	64.3	43.0	3.0	33.5

*f*_*cu*_ is the cube compressive strength, *f*_*c*_^'^is the axial compressive strength, *f*_*t*_ is the tensile strength, *E*_*c*_ is the modulus of elasticity.

**Table 3:** Measured mechanical parameters of the grout.

Categories		Performance index	Measured values
Fluidity	Initial	≥ 300 mm	310 mm
	30 min	≥ 260 mm	293 mm
Compressive strength	1 d	≥ 35 MPa	44.4 MPa
	3 d	≥ 60 MPa	63.2 MPa
	28 d	≥ 85 MPa	96.4 MPa
Vertical expansion rate	3 h	≥ 0.02%	0.08%
	From 3 to 24 h	0.02–0.50%	0.04%
Chloride ion content		≤ 0.03%	0.018%
Corrosion of reinforcement		0	0

**Table 4:** Mechanical properties of the steel reinforcement.

Diameter (mm)	D16	D8	D6
Types	HRB400	HPB300	HPB300
Area (mm^2^)	201	50	28
Yield strength (MPa)	454	357	329
Ultimate strength (MPa)	622	561	534
Elastic modulus (GPa)	205	197	198

**Table 5:** Geometric dimensions and related mechanical properties of the disc spring.

*D* (mm)	*d* (mm)	*t* (mm)	*H* (mm)	*h*_0_ (mm)	Maximum load *F* (kN)
35.5	18.3	2	2.8	0.8	5.19

The vertical expansion rate is a controlled index of GB/T 50448-2015 ‘‘Technical code for application of cementitious grout”^36^, which is a Chinese criterion regarding the grout.

### Test setup and loading procedure

**Figure 6:**
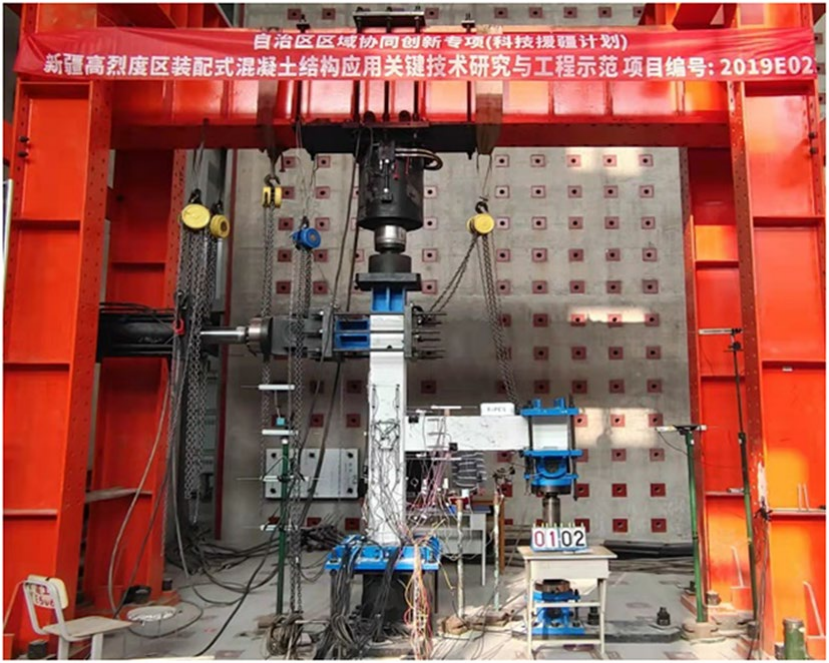
Test setup.

**Figure 7:**
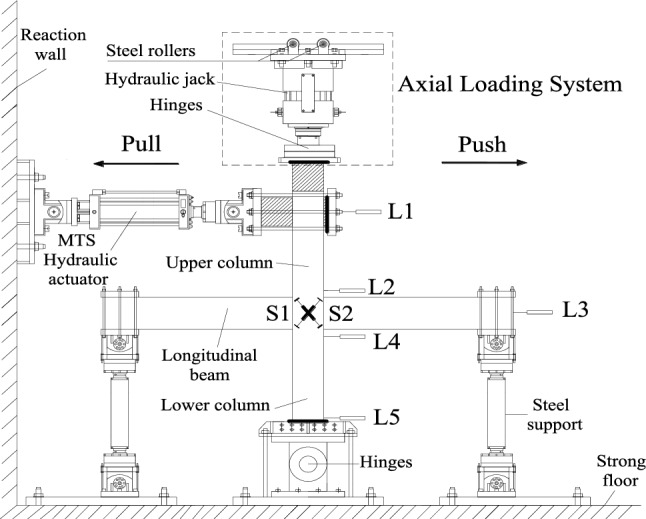
Schematic of the test setup.

**Figure 8:**
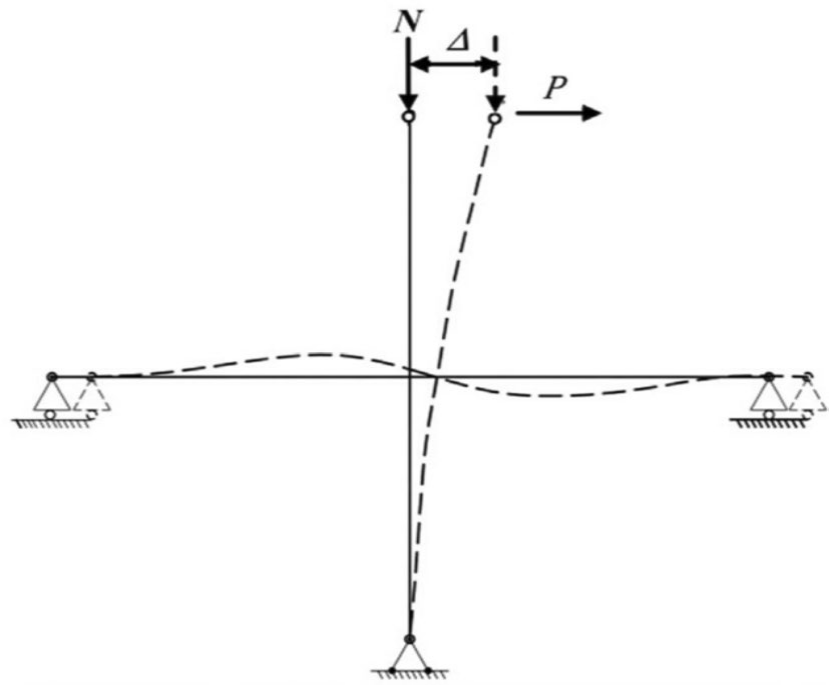
Deflected shape of the corner beam-column joint.

**Figure 9:**
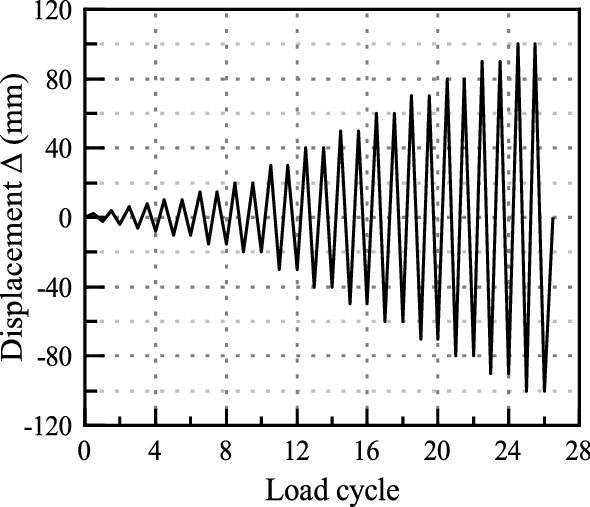
Cyclic loading procedure.

This test was carried out in the Laboratory of Xinjiang University. The low cycle reciprocating load test was utilized to evaluate the seismic performance of the specimens. The test equipment and boundary conditions are depicted in Fig. 6. The geometry of the specimen used is determined according to the span of the beam and column in the prototype structure, the travel of the actuator, and the distance between the ground anchors of the laboratory. Moreover, the geometry of the specimen is determined. For the purpose of simulating the boundary conditions, the vertical support of the free end of the beam is a double hinged rod, which allows the beam end to move horizontally and rotate freely without torque. A one-way hinge is attached to the solid ground at the bottom of the column to achieve zero bending moments, which can be considered the reverse bending point of the column. The column ends are subject to cyclic lateral and axial loads. Each test utilizes two linear variable displacement sensors (LVDTs), as shown in Fig. 7. Five LVDTs are positioned at varying heights on the member to assess its load‒displacement response L1–L5; four LVDTs numbered S1–S4 are set in the core region of the specimen to measure the shear deformation. Figure 8 depicts the lateral displacement shape of the member under the upper column end load, which resembles the real deformation state of reinforced concrete under seismic loading. The axial loading system, which can move horizontally with the top of the column, can achieve the second-order effect (*N-delta*) on the core region of the joint and the overall deformation. The design axial compression ratio of this test *μ* is 0.2 and 0.4, with the axial compression ratio *μ* defined as *μ* = *N/*(*A*·*f*_*c*_), where *N* is the vertical load exerted on the top of the column, *A* is the cross-sectional area of the column, and *f*_*c*_ is the axial compressive strength of the concrete. Before formal loading, the jacks with a 3000-kN bearing capacity should apply axial loads equal to 20% and 40% of the ultimate axial bearing capacity on the top of the column. An MTS horizontal actuator with a bearing capacity of 1000 kN and a maximum stroke of 600 mm is used to apply a cyclic load on the top of the column. When the load of the test piece reduces to approximately 85% of its peak load, the test stops. The cyclic loading system is shown in Fig. 9.

## Experimental results and analysis

### Crack distributions and failure patterns

**Figure 10:**
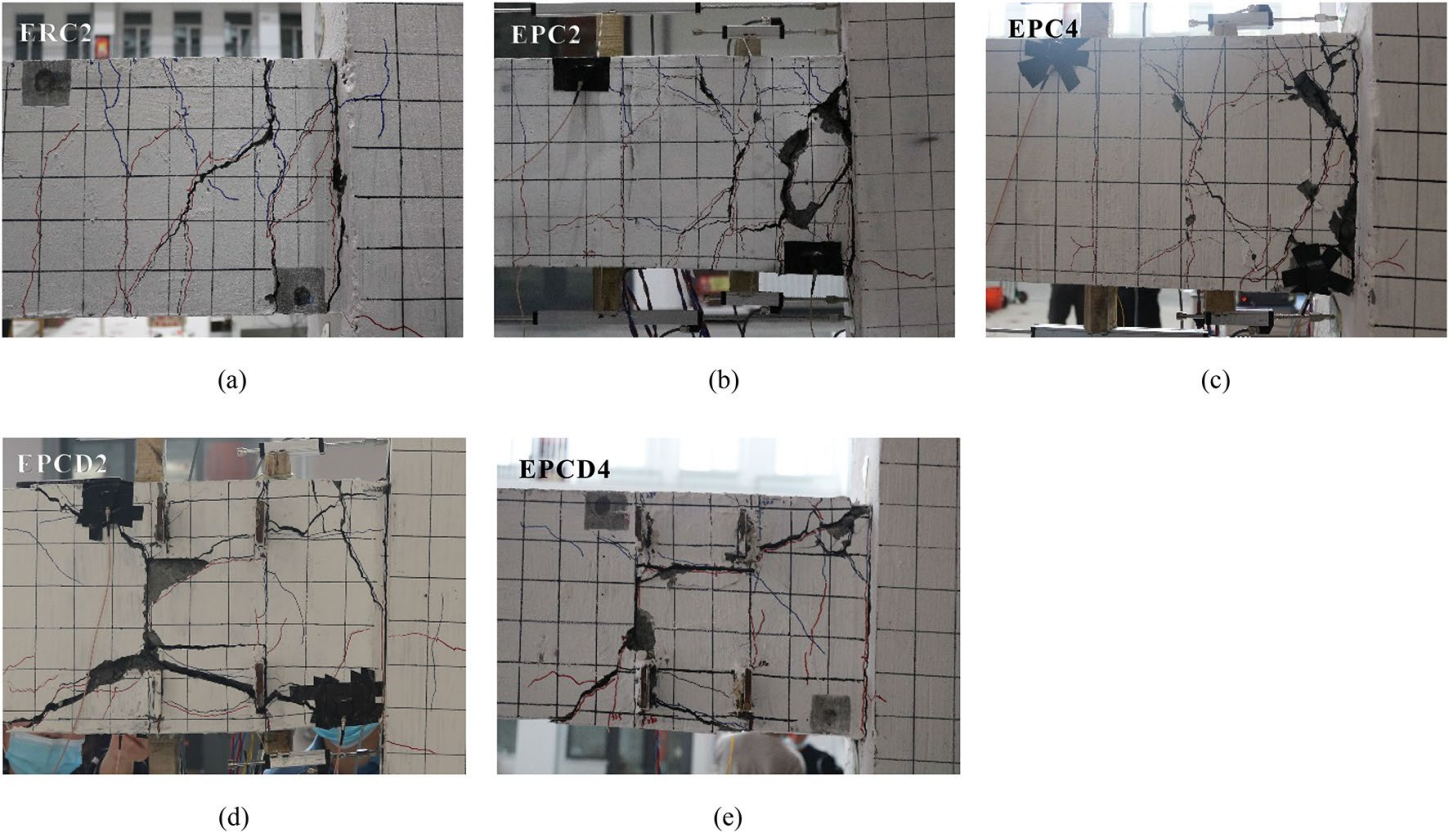
Crack distributions and failure patterns for the exterior joints (**a**) ERC2; (**b**) EPC2; (**c**) EPC4; (**d**) EPCD2; (**e**) EPCD4.

**Figure 11:**
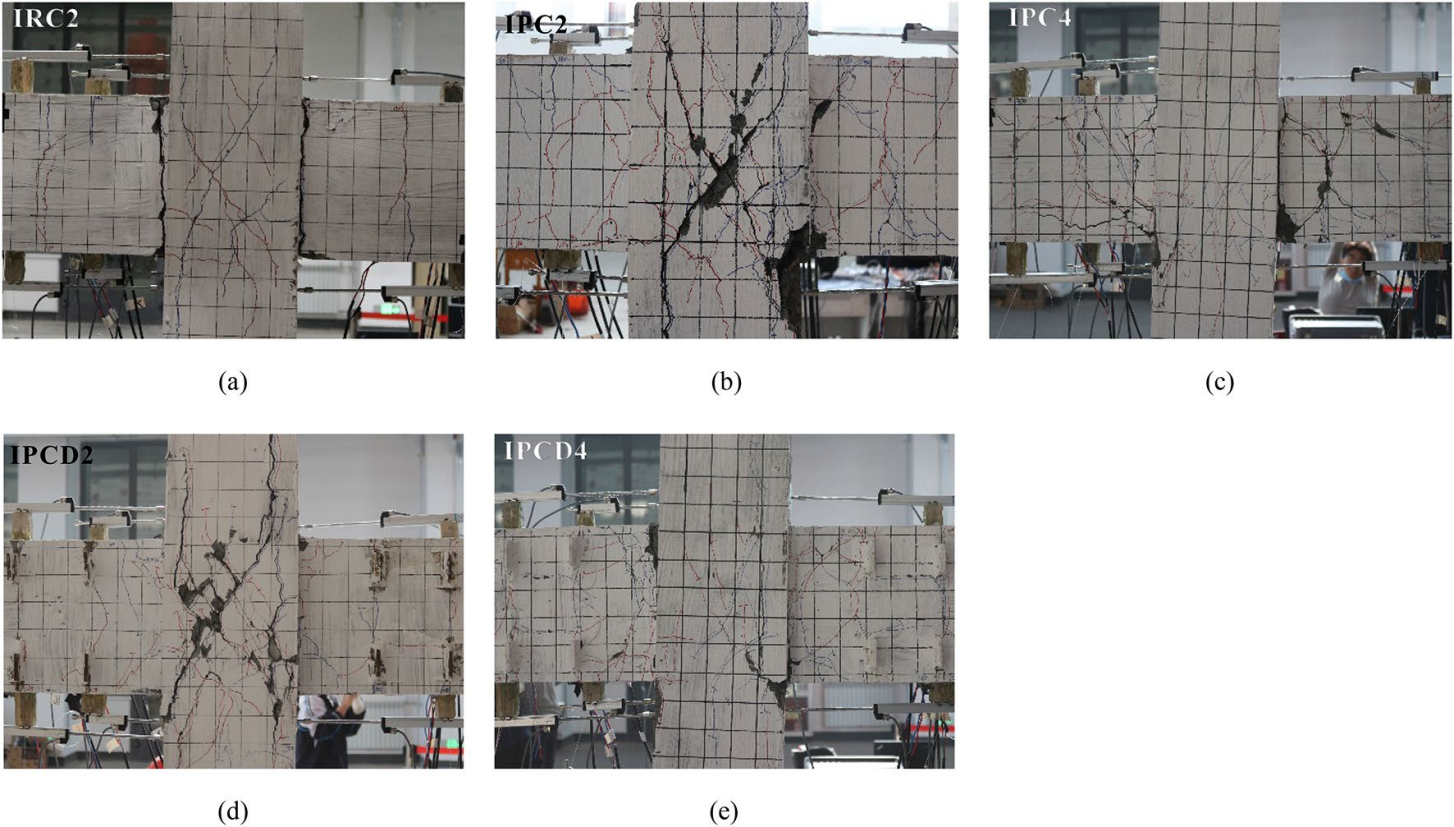
Crack distributions and failure patterns for the interior joints (**a**) IRC2; (**b**) IPC2; (**c**) IPC4; (**d**) IPCD2; (**e**) IPCD4.

Under a low cycle reciprocating load, the crack formations in the exterior joints and interior joints differ. Regarding the exterior joints, the cracks are primarily focused in the plastic hinge area at the beam end, and there are essentially no cracks in the core region of the joint; however, both the plastic hinge area and core region at the beam end of the interior joints are damaged to variable degrees. Figures 10 and 11 show the final failure mode of ten specimens. The detailed analysis is as follows.

1. *Monolithic joint* Specimens ERC2 and IRC2 have an identical crack distribution and mode of failure. Near the early stage of loading, vertical bending cracks occur at the end of the beam end. With an increasing loading displacement, it can be observed that the cracks evenly distributed at the beam end gradually increase and extend through, and the through cracks at the beam end are the most significant cracks. Due to the movement of the steel bar sliding in the plastic hinge area, longitudinal and oblique cracks appear at the exterior joint end of the ERC2 beam. Finally, with the development of cracks, serious concrete spalling occurs in the plastic hinge region. When the drift ratio of the interior joint IRC2 reaches 1.5%, microcracks emerge in the core region of the joint and then continue to grow. Nevertheless, the ultimate failure of the specimen is not caused by a significant number of microcracks in the core region but rather by the continual development of through cracks near the beam end. Failure modes of ERC2 and IRC2 are shown in Figs. 10a and 11a, respectively.
2. *Exterior joint* When the drift ratio is 0.4%, Specimens ERC2 and EPC2 exhibit bending cracks; then, the bending crack expands away from the beam end and produces a plastic hinge zone. The entire process of the EPC2 and EPCD2 damages is depicted in Supplementary Figs. A1 and A2, respectively. Due to the greater strength of the post-cast area of the precast members, the cracks in the plastic hinge region of the EPC2 beam end of the specimen are more concentrated, and the damage is more severe. The crack development process and failure features of the new specimen EPCD2 are different from those of the first two. During the loading process, the geometric depression of the post-pouring area and the geometric bulge at the end of the precast beam are meshed with each other, resulting in stress concentrations. This causes cracks to appear at the joint surface of the new and old concrete too early and then expand into oblique shear cracks through the post-pouring area. It is evident that most of the inelastic damage of Specimen EPCD2 is concentrated at the disc spring device so that it can be quickly inspected or reinforced after the actual earthquake. In the late stage of loading, except for two oblique main cracks, no further cracks occur during the final loading stage. As shown in Supplementary Fig. A2, the experiment was stopped when the lateral displacement ratio reached 4%. The bottom concrete of the beam was stripped, and the internal disc spring system and grouting sleeve were observed. It was discovered that the disc spring device and grouting sleeve were in good condition and that the reinforcement in the grouting sleeve did not slip. A section of reinforcement between the disc spring device and grouting sleeve was bent. It can be seen that the yielding position of the reinforcement originally happened here; however, this portion of reinforcement did not have visible necking. The failure process of Specimens EPC4 and EPCD4 is comparable to that of Specimens EPC22 and EPCD2. However, the increase in the axial compression ratio delays the emergence of cracks and inhibits their development. In general, the most significant damage to the side joints is always centered in the plastic hinge region at the beam end, particularly at the beam-column interface and disc spring region. All specimens fail due to the yielding of the longitudinal bars and the crushing of concrete at the end of the beam, indicating an optimal hinge mechanism (Fig. 10a–e).
3. *Interior joint* The interior joints show different failure modes. At the beam ends of Specimens IRC2 and IPC, uniformly distributed bending cracks emerge during the early stages of stress. When the drift ratio reaches 1%, the beam-column interface is penetrated. After the drift ratio reaches 1.5%, oblique fractures emerge in the core region of the joint, and the gradually increasing oblique cracks create cross cracks. The vertical bending cracks at the beam end of the IPC2 specimen are more concentrated, and the number of inclined cracks in the core region is greater. Intriguingly, the width of the oblique crack in the core region of IRC2 of the specimen did not significantly increase; however, the gap between the beam and column interface repeatedly opened and closed under cyclic loading, and the width continued to increase, resulting in the ultimate failure of the specimen. Supplementary Figures A3 and A4 show the damage process of Specimens IPC2 and IPCD2. The failure modes of Specimens IPC2 and IPCD2 are similar, and the rise in the post-pouring area strength distinguishes their failure modes from those of the monolithic specimen IRC2. During the loading procedure, cracks continued to propagate toward the core region. After the drift ratio reached 2.5%, the cross crack in the core region developed into the main crack, and finally, the specimen failed under the combined effects of the shear deformation in the core region and bending deformation at the beam end. Supplementary Figure A4 reveals that when the displacement ratio reaches 5%, there are still a few cracks at the beam end of Specimen IPCD2, indicating that the disc spring device has played a role throughout the entire testing procedure; compared to Specimen IPC2, the damage of the new joint IPCD2 is more concentrated, and the shear deformation of the core area is more pronounced. Specimens IPC4 and IPCD4 with greater axial loads exhibited the same bending failure mode as the monolithic specimen IRC2, with the longitudinal reinforcement at the beam end being the first to yield. The failure modes of the same specimen under different axial compression ratios indicate that increasing the axial load can increase the shear capacity of the core area. Figure 11c,e also confirm that the increase in the axial stress slows the development of inclined cracks in the joint region. As a result of the complicated stress conditions of the axial compression, shear, and bending moment coupling in the core area of the beam-column joint, damage in the joint area of the interior joint appears to varying degrees (Fig. 11a–e).

### Hysteretic characteristics

**Figure 12:**
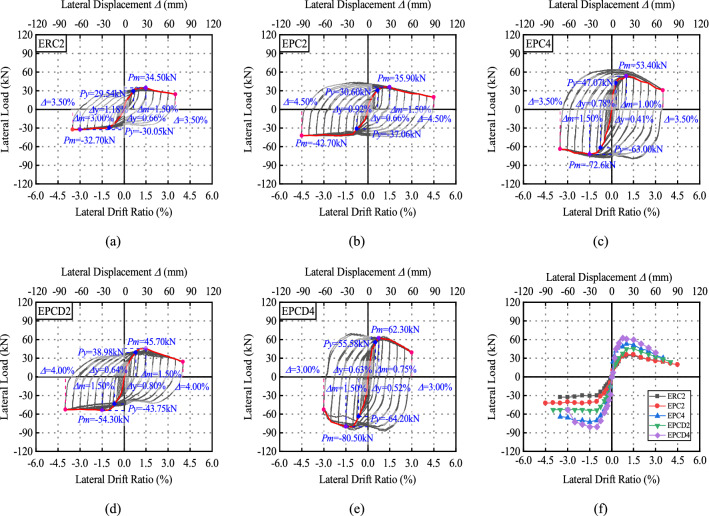
Hysteretic and envelope curves for exterior connections.

**Figure 13:**
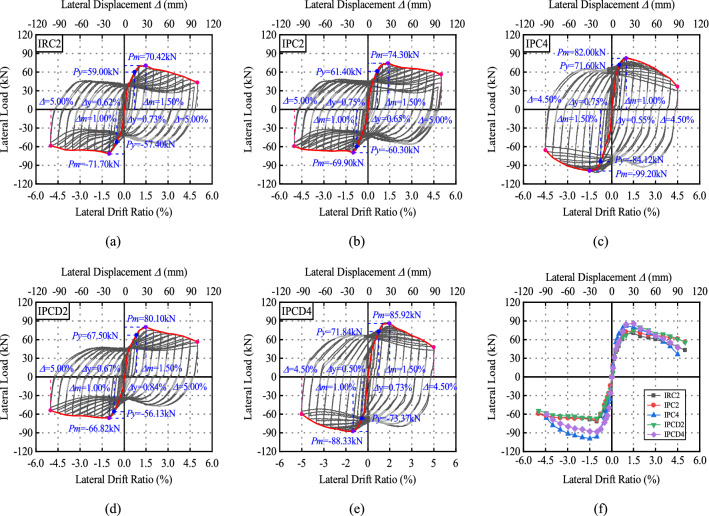
Hysteretic and envelop curves for interior connections.

**Figure 14:**
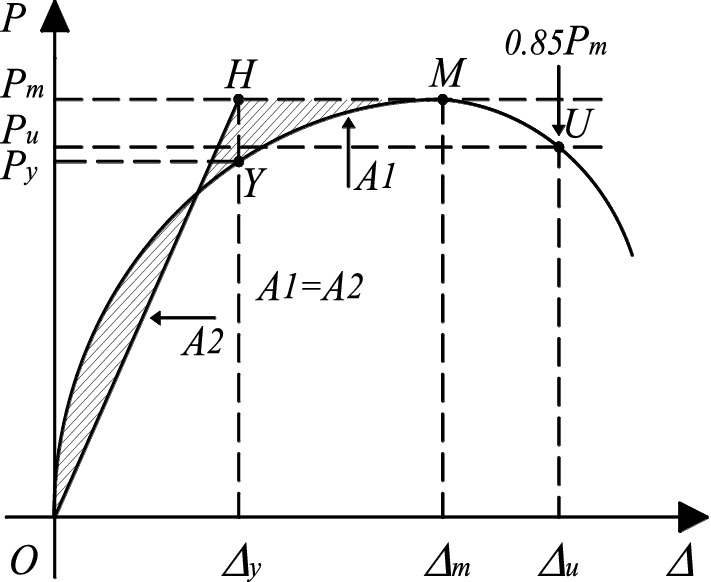
Method used to define the yield displacement.

Figures 12 and 13 depict the load‒displacement curve and envelope curve of 10 test specimens. To quantitatively analyze the seismic performance of the specimen, the envelope curve is used to determine the yield point, peak point, and limit point, as well as the lateral displacement ratio and lateral force corresponding to each characteristic point. The yield point is defined by the equivalent elastic‒plastic energy criterion proposed by Park^37^. As depicted in Fig. 14, when the areas *S*_1_ and S_2_ are equal, the location of point *H* can be established. A vertical line perpendicular to the horizontal axis is drawn through Point H, and the point at which it intersects the envelope curve is the yield point; the point at which the load drops to 85% of the peak load is defined as the ultimate point. Table 6 summarizes the yield displacement ratio Δ_y_, yield load *P*_y_, peak load *P*_m_, related lateral displacement ratio Δ_m_, ultimate lateral displacement ratio Δ_u_, and displacement ductility coefficient of Specimen *μ*. *μ* is calculated as the ratio of the ultimate drift ratio to the yield drift ratio, which reflects the plastic deformation capacity of the specimen.

**Table 6:** Seismic performance indices of the test specimens.

Specimen	Δ_y_ (%)	*P*_*y*_ (kN)	Δ_m_ (%)	*P*_*m*_ (kN)	Δ_u_ (%)	Ductility coefficient *μ*	Failure mode
ERC2	0.77	28.46	1.50	32.20	3.50	4.56	FF
EPC2	0.74	32.38	1.50	38.65	3.01	4.09	FF
EPC4	0.59	52.72	1.50	62.15	2.34	3.97	FF
EPCD2	0.76	42.20	1.50	50.00	3.68	4.85	FF
EPCD4	0.60	59.90	1.00	70.95	2.77	4.62	FF
IRC2	0.67	58.38	1.00	70.35	3.91	5.84	FF
IPC2	0.82	61.01	1.00	71.30	4.23	5.16	SF
IPC4	0.63	75.44	1.50	89.65	2.94	4.65	FF
IPCD2	0.70	63.25	2.00	72.50	4.68	6.66	SF
IPCD4	0.64	72.44	1.50	86.70	3.14	4.89	FF

*μ* is the displacement ductility coefficient, *FF* is the flexural failure, *SF* is the shear failure.

Because the positive and negative symmetries of the load‒displacement curve are poor, the positive and negative directions of each load‒displacement curve are used as the yield point and peak point, respectively, for reference purposes. At the initial stage of loading, the load‒displacement curve for the monolithic specimens ERC2 and IRC is linear elastic, the residual deformation is small, and the cracks develop uniformly. With the loading displacement, the envelope area of the hysteretic curve gradually increases, and the strength of the specimen slowly decreases after reaching the peak load, indicating that the monolithic specimen has good hysteretic performance. The yield load, peak load, and lateral displacement ratio corresponding to the characteristic load of Specimens EPC2 and IPC2 are comparable to those of the monolithic specimen, showing that this assembly mode is more reliable. The new specimens EPCD2 and IPCD2 show more stable hysteretic characteristics. The peak loads of the two specimens are 45.70 kN and 80.1 kN, which are increased by 32.46% and 13.75%, respectively, compared to the monolithic specimens of ERC2 and IRC2 (35.9 kN and 74.3 kN). Comparing the three types of joints, it is found that the displacement ratio corresponding to the yield point of the new joint with an integrated built-in disc spring device is the greatest, showing that the existence of the disc spring device delays the emergence of the yield point. Observing the load‒displacement curve of each specimen reveals that the lateral displacement of the new joint reduces more slowly than other joints during the process of lateral force unloading in each cycle, and the residual displacement is the smallest when the joint is completely unloaded. Before the displacement ratio hits 1%, the load‒displacement curve of the new joint is closer to the return curve, indicating that the disc spring device can participate in the work in the early stage of the test. As a result of the longitudinal bar slip at the beam end, the unloading process of the load‒displacement curve of the constructed specimen is extremely steep and then extremely flat. This phenomenon is more apparent in Figs. 12b,c and 13b,c, demonstrating that there is more slip-in conventional prefabricated joints, which also indirectly confirms that the disc spring device improves reinforcing slip. In Fig. A2, the internal reinforcement condition is directly detected after the test, and it is also determined that the slip of the reinforcement is minimal. When the axial compression ratio is 0.4, the failure mechanisms of EPC4, EPCD4, IPC4 and IPCD4 are identical, and the load‒displacement curves have a high degree of similarity. The increase in the axial compression ratio does not change the failure mode of the exterior joint specimen, but it changes the failure mode of the interior joint specimen from the shear failure in the core region to the bending failure, and the load‒displacement curve changes from an “S” shape (Fig. 13b,d). The original anti-“S” shape (Fig. 13b,d) develop into a fuller shuttle shape (Fig. 13c,e), indicating that the increase in the axial compression ratio can not only improve the bearing capacity of the specimen but also change its failure mode. During the test, because the core region of IPC2 and IPCD2 was sheared and broken, the pinch effect was more pronounced. The increase in the axial compression ratio limits the development of cracks on the column, resulting in the bending failure of the specimen formed by the plastic hinge at the beam ends so that the load‒displacement curves of Specimens IPC4 and IPCD4 are more similar to those of IRC2. At a later stage of loading, all specimens demonstrated an increase in the pinch effect and more residual deformation.

From the envelope curves in Figs. 12f and 13f, and Table 6, it is more intuitive to observe the differences in the bearing capacity, strength degradation, and ductility of each specimen, and it can be observed that ten specimens have obvious yield stages. When the axial compression ratio is 0.2, the envelope curves of the monolithic specimen and the ordinary precast specimen are not significantly different. The yield lateral displacement ratio and peak lateral displacement ratio of Specimens ERC2, EPC2, and EPCD2 are similar, but the yield load (42.2 kN) and peak load (50.00 kN) of the new specimen EPCD2 with the built-in disc spring device are increased by 48% and 55%, respectively, in comparison to ERC2. The interior joint also has a similar lifting effect, which is induced by the disc spring increasing the local stiffness of the beam end. Compared to the monolithic specimen ERC2 (IRC2), the ductility coefficient of the ordinary precast specimen EPC2 (IPC2) is found to slightly decrease, and the ductility coefficient of the new, precast specimen EPCD2 (IPCD2) slightly increases, indicating that the disc spring is beneficial to improving the ductility of the specimen. The increase in the axial load can improve the bearing capacity of the specimen. In comparison to the interior joint, the increase in the exterior joint is more noticeable. Taking Specimens EPC2 and EPC4 as examples, the peak load of the latter is increased by 61% compared to that of the former. The coefficient of the displacement ductility diminishes as the axial load increases because, under a greater axial load, the specimen rapidly degenerates to less than 85% after reaching the peak load.

### Strength and stiffness degradation

**Figure 15:**
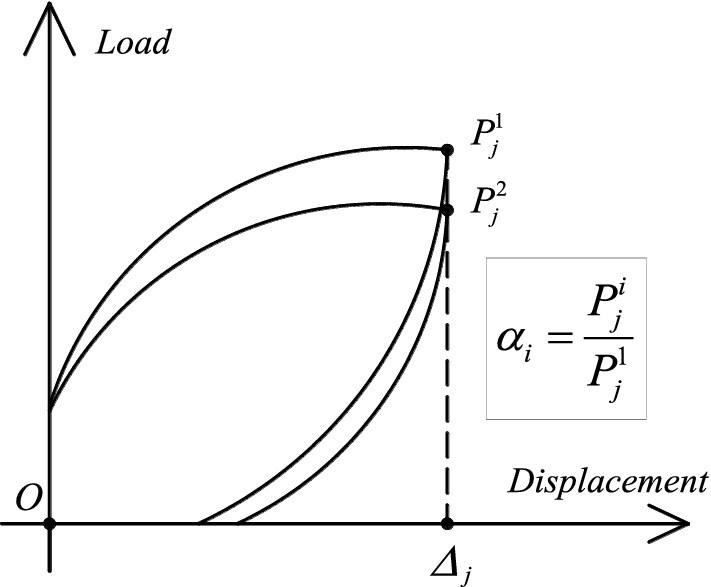
Definition of the strength degradation ratio.

**Figure 16:**
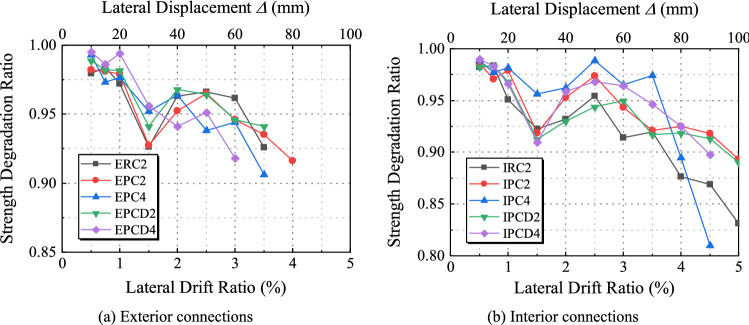
Strength degradation ratio.

Under a given lateral drift ratio, the strength degradation may occur due to damage accumulation during multiple loading cycles. The strength degradation ratio *α* is defined as the ratio of the bearing capacity under the *i*th (*i* = 2) cycle to the bearing capacity under the first cycle, as depicted in Fig. 15. Figure 16a,b depict the strength degradation ratio of the exterior joints and interior joints, respectively. The 10 specimens were in the elastic range at the start of the load test, and the strength degradation was not readily apparent. The strength degradation curve abruptly reduces when the lateral displacement ratio is 1.5% and then climbs slightly between 1.5 and 2.5%. The position of the abrupt decrease in the curve corresponds to the position where the specimen reaches the peak load. During this process, the reinforcement yields, and the concrete damage accumulates. From the strength degradation curve of the specimen with an axial compression ratio of 0.2, it can be seen that when the lateral displacement ratio reaches 5% from 3.5%, the *α* of Specimen ERC2 decreases by 9.8%, whereas the *α* of Specimens EPC2 and EPCD2 decrease by only 4.3% and 5.4%, respectively. The specimens with an axial compression ratio of 0.4 exhibited a stronger strength degradation effect as the lateral displacement ratio increased from 3.5 to 4.5%, and the *α* of IPC4 and IPCD4 decreased from 0.97 and 0.95 to 0.81 and 0.9, respectively. Comparing the strength degradation curves of all specimens reveals that the strength degradation ratio of Specimens EPCD2, EPCD4, IPCD2 and IPCD4 is comparatively low, and the degradation curve is more gradual, indicating that the disc spring device has a certain mitigation effect on the strength degradation. Under the action of a greater axial load, this mitigation effect becomes more pronounced, and the degradation curves of Specimens IPC4 and IPCD4 clearly illustrate this phenomenon. The *α* of the exterior joint exceeds 0.9, while the *α* of the interior joint exceeds 0.8. All specimens meet the acceptance standard that the strength degradation coefficient should not be less than 0.75 specified in ACI 374.1-05^38^.

**Figure 17:**
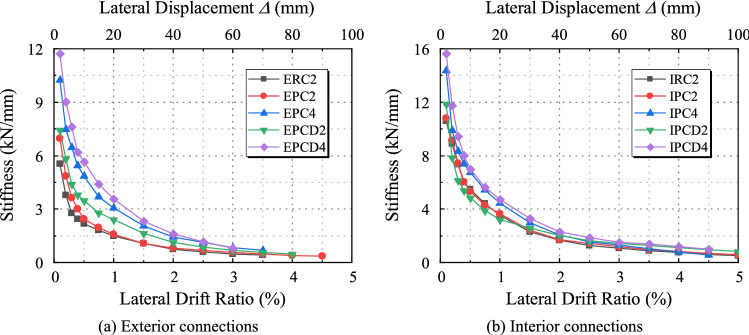
Stiffness degradation.

The degradation of the stiffness is another important parameter that evaluates the seismic response of the structures since it indicates the cumulative damage induced by the seismic loads. If the joint stiffness significantly decreases during an earthquake, a small load will lead to a great deformation of the structure and cause it to become unstable; hence, stiffness degradation is crucial when evaluating the seismic performance of the structure. The secant stiffness is utilized to compare the decrease in the specimen stiffness. The secant stiffness is defined as the slope of the line between the load point and the origin that corresponds to the maximum lateral displacement ratio of each cycle^39^. The secant stiffness of each test piece is depicted in Fig. 17. Due to the buildup of the specimen damage, it can be observed that the stiffness diminishes as the lateral displacement ratio increases. Specimens EPC2 and IPC2 exhibit a similar degradation trend to Specimens ERC2 and IRC2, indicating that the precast connectors with full grouting sleeves exhibit the same stiffness degradation performance as cast-in situ connectors. The initial stiffness of the specimen subjected to a stronger axial load is much greater than that of Specimens EPC2 and IPC2, among which the local stiffnesses of Specimens EPCD4 and IPCD4 are increased. Thus, the initial stiffness is the highest. However, with the increase in the displacement, the concrete fractures and steel bars yield, making the stiffness degradation of the specimen with a high axial compression ratio even more pronounced. Before the displacement ratio reaches 1%, the stiffness degradation curve of the new prefabricated joint becomes steeper and then flatter. This is because the concrete section at the disc spring device at the end of the new joint beam is smaller, resulting in a reduced tolerance for damage. Gradually, the disc spring device plays a function in the process of ongoing concrete degradation. Even if the concrete significantly cracks, the stiffness of the new prefabricated joint is still not completely lost. It maintains some rigidity until the end of the test, it still retains a certain stiffness, and the location of the concrete damage is predictable. Regarding the monolithic specimen and regular precast specimen, the permanent damage of concrete continues to increase with an increasing displacement ratio, and the stiffness of the specimen is almost entirely lost by the end of the test. After a displacement ratio of 2.5% occurs, the stiffness of the four new prefabricated joints is always greater than that of the monolithic specimen and the conventional prefabricated specimen due to the outstanding damage tolerance of the disc spring device. The above results demonstrate that the disc spring can not only increase the initial stiffness of the member but also reduce the stiffness degradation and has a major influence on preventing the collapse of the structure during an earthquake.

Due to the varying axial compression ratio and internal structure of the specimen, the degree of the strength and stiffness degradation varies throughout the entire testing procedure. The decline of the strength and stiffness of the monolithic specimen is due to the constant buildup of concrete damage and longitudinal reinforcement deformation; the existence of a full grouting sleeve in ordinary precast specimens increases the damage area of concrete and increases the concentration of the longitudinal reinforcement. However, the full grouting sleeve can improve the local strength and stiffness of the beam end, and finally its strength and stiffness degradation curve is similar to that of the monolithic specimen. The new, precast specimen is built with a full grouting sleeve and disc spring device, so the strength degradation of the specimen is more obvious in the early stage of loading. In the later stage, the disc spring device with a good deformation ability fully played its role, alleviating the degradation rate of strength and stiffness, and finally the strength and stiffness degradation curve of the new joint was slightly higher.

### Energy dissipation and damping ratio

**Figure 18:**
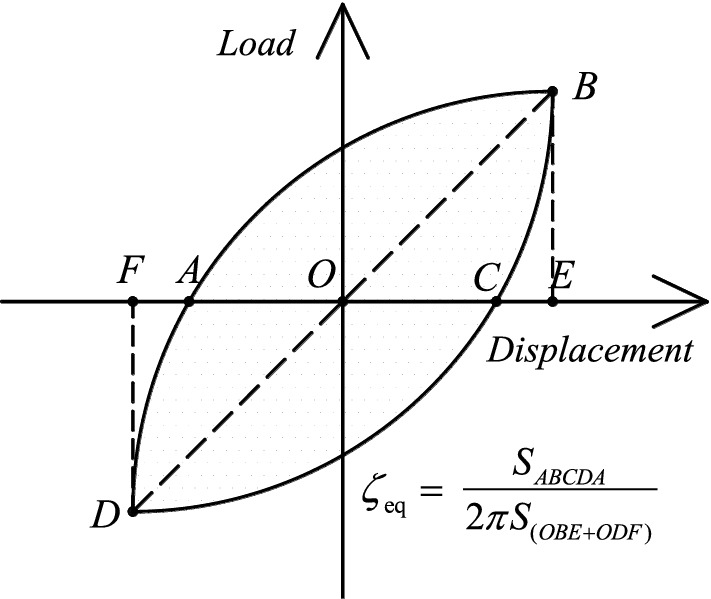
Definition of the equivalent viscous damping ratio.

**Figure 19:**
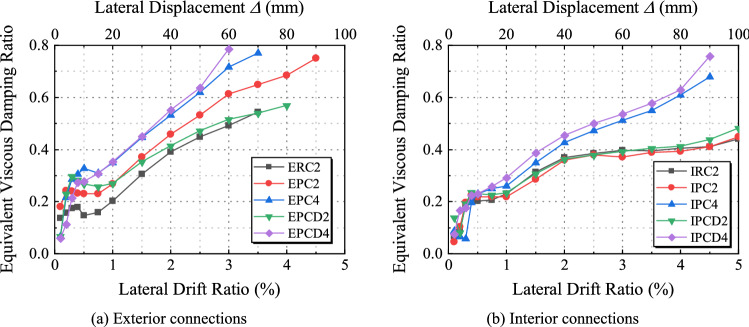
Equivalent viscous damping ratio.

Cumulative energy dissipation is a crucial metric for determining the energy dissipation capabilities of a structure. The energy dissipation of each cycle is represented by the area around the cyclic hysteresis loop, and the cumulative energy dissipation is defined as the sum of the energy dissipation of the continuous cycle. The equivalent viscous damping ratio^40^, *ζ*_*eq*_, is depicted in Fig. 18 and derived using formula (1). *S*_ABCDA_ represents the area surrounding the hysteresis loop for a given displacement, whereas S _(OBE+ODF)_ is the sum of the areas of the right tri-ratio OBE and ODF. The size is controlled not only by the area of the hysteresis loop but also by the fullness of the hysteresis loop, as shown in Fig. 19. The smaller the area enclosed by the hysteresis curve is, the more severe the pinch, and the smaller *ζ*_*eq*_ is; therefore, it can be utilized as a further crucial index to evaluate the energy consumption capacity of the structure.

1 $$\zeta_{eq} = \frac{{S_{ABCDA} }}{{2\pi (S_{OBE} + S_{ODF} )}}.$$

**Figure 20:**
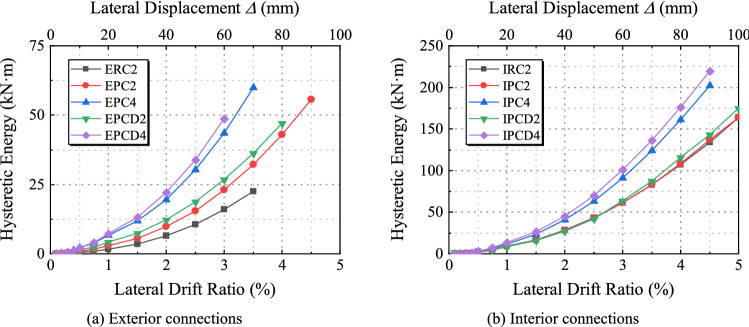
Cumulative energy dissipation curves.

Before the lateral displacement ratio approaches 0.5%, the energy dissipation capacity of the specimen is relatively low; however, it considerably increases upon entering the elastoplastic stage. Figures 19a and 20a show that *ζ*_eq_ and the cumulative energy consumption of the exterior joint specimens continue to increase. Comparing the cumulative energy consumption of ERC2, EPC2, and EPCD2 when the lateral displacement ratio is 3.5% reveals that the cumulative energy consumption of EPCD2 is the highest, which is 61% and 13% more than those of Specimens ERC2 and EPC2, respectively, and there is a similar increase compared with its counterpart. Figure 19b shows that the *ζ*_eq_ values of IPC2 and IPCD2 are roughly the same. Due to different degrees of shear deformation in the core area of both, the pinch effect of the hysteresis curve is more severe in the late stages of the test loading. Figure 20b shows that the cumulative energy dissipation of the two is relatively low, which is governed by the failure mode. In Fig. 19, the horizontal section of the curve emerges before the displacement ratio reaches 1% because the specimen is in an elastic state, and the shape of the load‒displacement curve under each cycle has the same shape. Figures 19 and 20 also show that the cumulative energy consumption of the specimen increases as the axial load increases. In contrast, the increase in the new joint with the disc spring device is more evident. When the ratio of lateral displacement is 4.5%, the cumulative energy consumption of Specimen IPCD4 is 60% and 53% higher than those of IPC2 and IPCD2, respectively. From the analysis of the slope of the energy dissipation curve, it is found that the slope of the new specimen with a built-in disc spring is larger, and if the test continues, the cumulative energy dissipation will be greater, indicating that the characteristics of high deformation and high load characteristics of the disc spring significantly enhance the cumulative energy dissipation capacity of the specimen.

### Shear deformation of the joint core region

**Figure 21:**
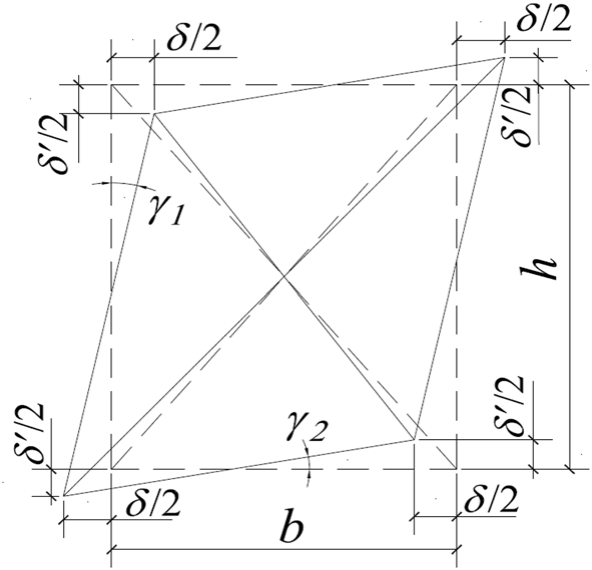
Evaluation method of the shear deformation.

**Figure 22:**
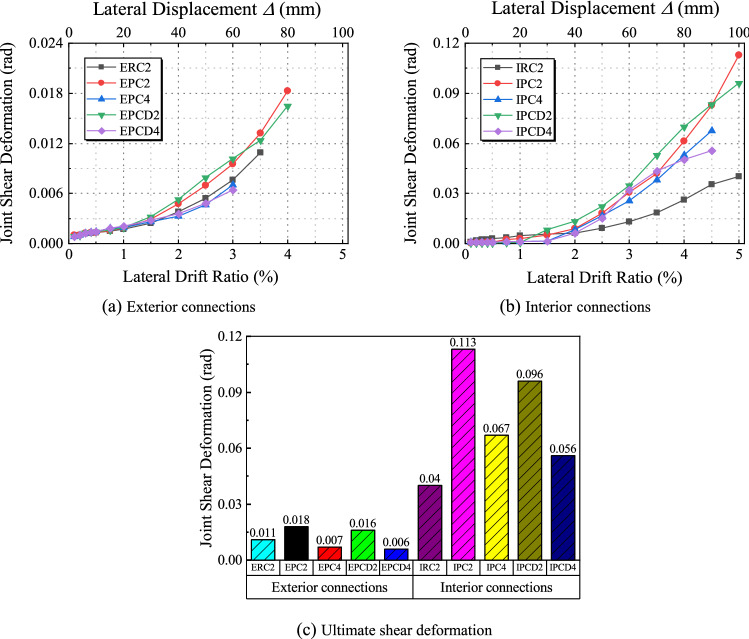
Shear deformation of the joint core region.

The shear deformation in the vicinity of the joint region is also a crucial indicator of the joint performance. According to Figs. 21 and 22, the shear deformation of the joint region *γ* is assessed in this work. The definition of *γ* is therefore defined as follows:

2 $$\gamma = \gamma_{1} + \gamma_{2} = \left( {\left| {\delta_{1} + \delta_{1}^{^{\prime}} } \right| + \left| {\delta_{2} + \delta_{2}^{^{\prime}} } \right|} \right)\frac{{\sqrt {b^{2} + h^{2} } }}{2bh},$$

where *δ*_1_, $\delta_{1}^{^{\prime}}$, *δ*_2_ and $\delta_{2}^{^{\prime}}$ are the relative deformations in the horizontal and vertical directions, respectively; and *b* and *h* are the horizontal gauge distance and vertical gauge distance of the joint area, respectively.

Figure 22 shows the average shear deformation of the node area of each specimen under different lateral displacement ratios. The ultimate shear deformation of the joint region of the exterior joint is relatively minimal, as seen in Fig. 22c. The ultimate shear deformation of the joint area of Specimen EPC2 is the largest, only reaching 0.018, because the deformation of the exterior joint is concentrated at the beam end, and there is almost no crack in the joint area. However, some conclusions can still take certain implications from the curve. The ultimate shear deformation of Specimens EPCD2 and EPCD4 is less than that of Specimens EPC2 and EPC4, and an increase in the axial load can also lessen the shear deformation. This phenomenon is more apparent in Fig. 22b. Before the lateral displacement ratio of 0.5%, the specimen does not yield and exhibits a similar, tiny shear response that gradually increases. Before the lateral displacement ratio reaches 3%, due to the larger initial stiffness of the beam ends of IPCD2 and IPCD4, the increasing rate of shear deformation is more pronounced and then gradually becomes progressively gentler under the influence of the disc spring. The ultimate shear deformation is reduced by 17% and 21% compared to IPC2 and IPC4, respectively. In Fig. 22b, it is observed that the ultimate shear deformations of Specimens IRC2, IPC4, and IPCD4 are limited because their ultimate failure modes are the bending failures, and the damage in the joint region is relatively light. Comparing the shear deformation curves of Specimens IPC2, IPCD2, IPC4, and IPCD4, it is discovered that the curves of the ordinary prefabricated specimens IPC2 and IPC4 gradually become steeper as the loading displacement increases. The shear deformation increments of the joint areas of IPCD2 and IPCD4 of the new specimen gradually decreases. Moreover, the curve becomes gentler, which is attributed to the excellent deformation ability and self-resetting performance of the disc spring device. The increase in the axial load prevents the development of cracks in the joint region. When the lateral displacement ratio is 4.5%, the shear deformation of Specimens IPC4 and IPCD4 is 67% and 73% less than that of specimens IPC2 and IPCD2, respectively, with the ultimate shear deformation of Specimen IPCD4 being just 0.056 rad. It is evident that the application of a disc spring device and the increase in axial load significantly reduce the shear deformation in the joint region.

## Conclusion

In this study, a new type of joint is designed, in which a beam-end disc spring device is incorporated. The seismic performance of ten 1/2-scale joints is examined and evaluated. The primary objective of this paper is to explore the seismic performance of new precast joints and to explore the crack distribution characteristics and failure modes of specimens under different axial compression ratios. Based on the analysis of the test phenomena and the discussion of the test results, the following conclusions can be drawn.

1. Compared with conventional precast concrete joints, the bearing capacity, displacement ductility, cumulative energy consumption, and shear deformation of the new prefabricated concrete joints have been enhanced, and their seismic performance is superior to that of the monolithic specimen, indicating that the assembly mode adopted in this paper meets the design requirements of “equivalent cast-in-place”.
2. The displacement ductility coefficient of ordinary precast specimens is slightly lower than that of integral cast-in-place specimens, but the ductility coefficient of new prefabricated joints is higher than that of integral cast-in-place specimens, indicating that the ductility of precast specimens is greatly improved by the disc spring device at the beam end.
3. The equivalent viscous damping coefficient of the prefabricated specimen is nearly the same as that of the whole specimen, but the cumulative energy consumption is greater than that of the monolithic specimen, and the newly constructed joint has the highest energy consumption capacity.
4. At the end of the test, the disc spring device was not significantly damaged. During an earthquake, the accumulation of permanent damage to concrete may result in the total loss of the seismic capacity of the structure. During the test, it was discovered that even if the concrete of the new prefabricated specimen was significantly damaged, the specimen still retained a certain amount of strength and stiffness due to the existence of the disc spring device.
5. The shear deformation of the newly fabricated joint is greater than that of the ordinary prefabricated specimen in the early stage and less than that of the conventional prefabricated specimen in the late stage, indicating that the disc spring device can alleviate shear deformation. The larger the displacement ratio is, the more apparent this impact becomes.
6. The initial stiffness, bearing capacity, and energy consumption of the specimen will significantly increase as the axial load is increased. However, after the peak load occurs, the strength of the specimen rapidly declines, resulting in a significant reduction in the ductility coefficient. At the same time, an increase in the axial load changes the failure mode of prefabricated joints from shear failure to flexural failure, indicating that a larger axial load can inhibit the crack propagation in the joint area; therefore, the influence of the axial load on the overall deformation of the structure must be fully accounted during structural designs.

## Supplementary Information

Supplementary Figures.

## Data Availability

The datasets used and/or analyzed during the current study are available from the corresponding author on reasonable request.
