# Supplementary material for: Seismic performance of a new precast concrete frame joint with a built-in disc spring
Source: Sci Rep. 2023 Apr 1;13:5334. doi: 10.1038/s41598-023-32447-1 (PMC10067826; doi:10.1038/s41598-023-32447-1)
Supplement: Supplementary file 1 — Supplementary Figures. [file 41598_2023_32447_MOESM1_ESM.docx]

# Appendix

| 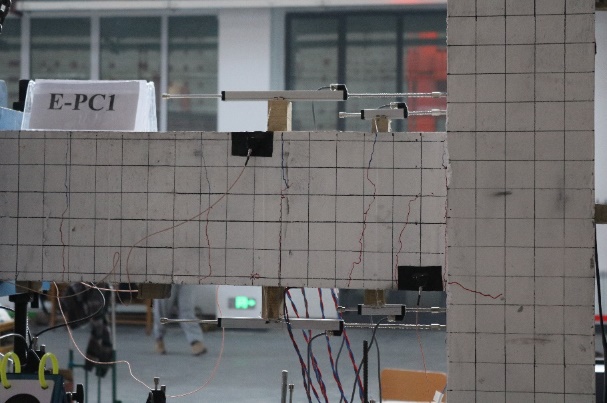 | 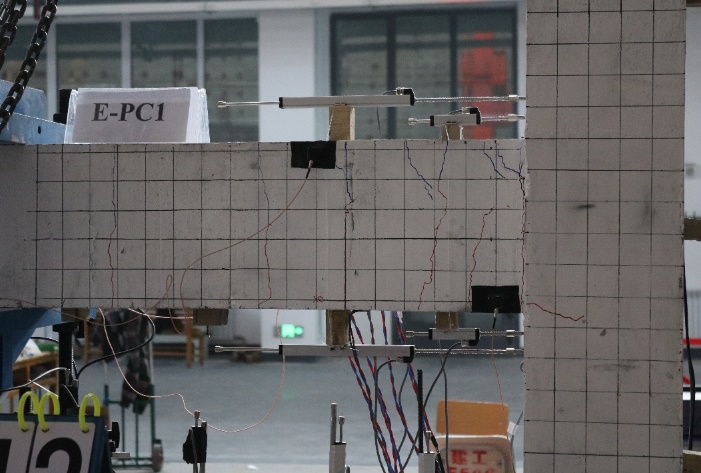 | 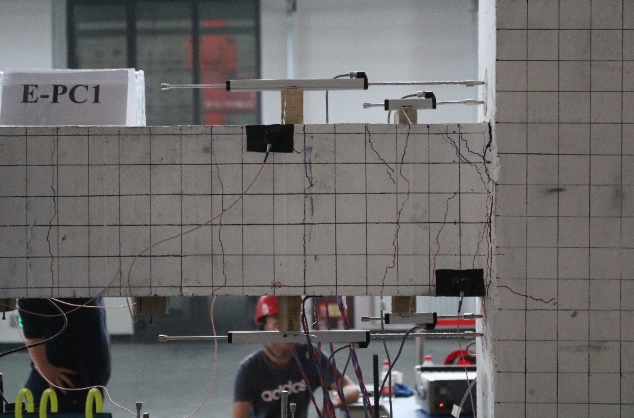 | 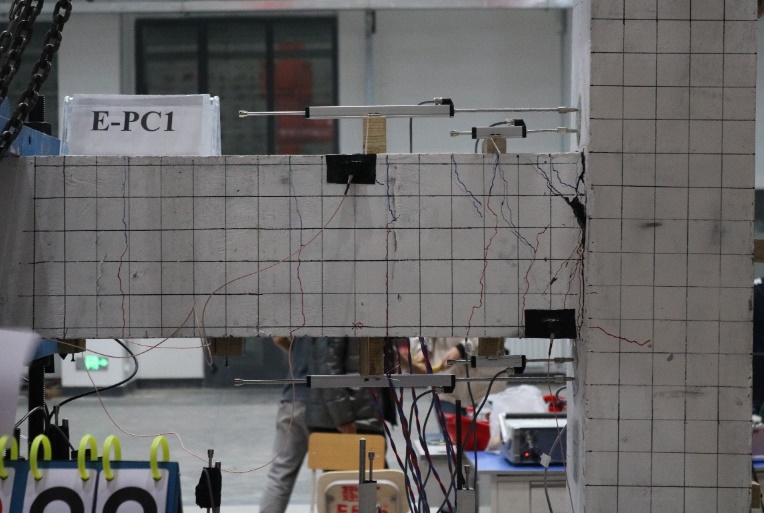 |
| --- | --- | --- | --- |
| *Δ*=1.0% | *Δ*=1.5% | *Δ*=2.0% | *Δ*=2.5% |
| 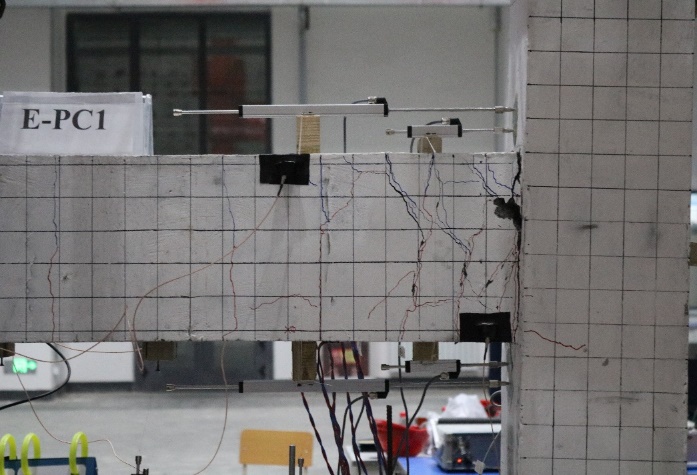 | 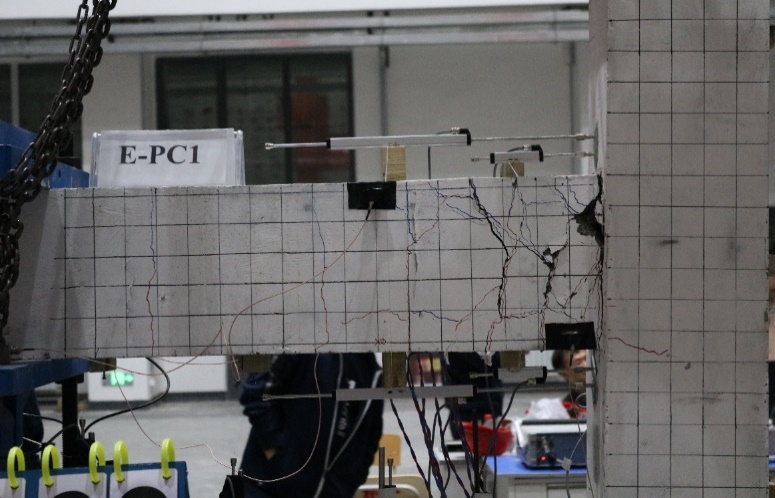 | 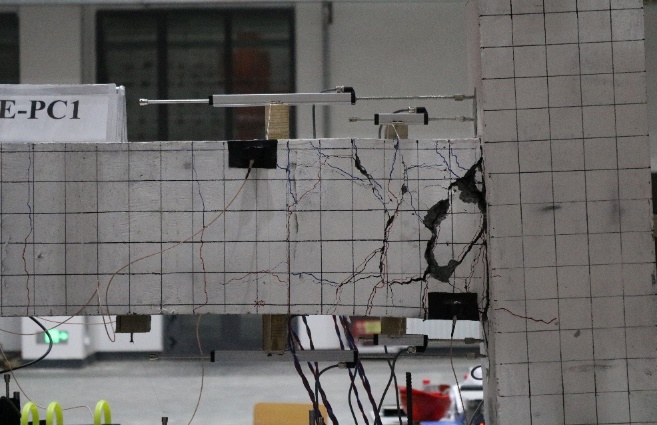 | 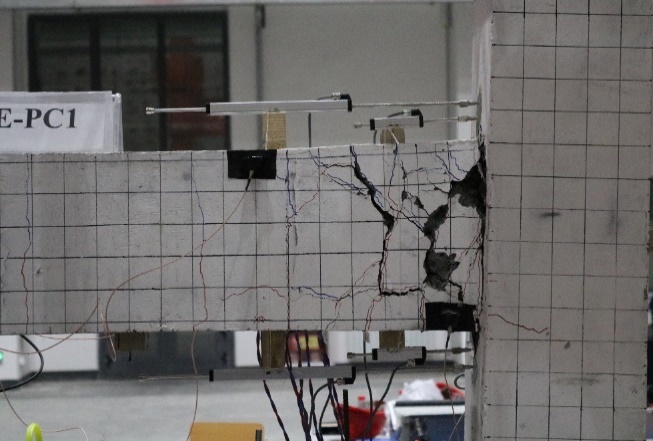 |
| *Δ*=3.0% | *Δ*=3.5% | *Δ*=4.0% | *Δ*=4.5% |

**Fig. A1.** Damage process of EPC2.

| 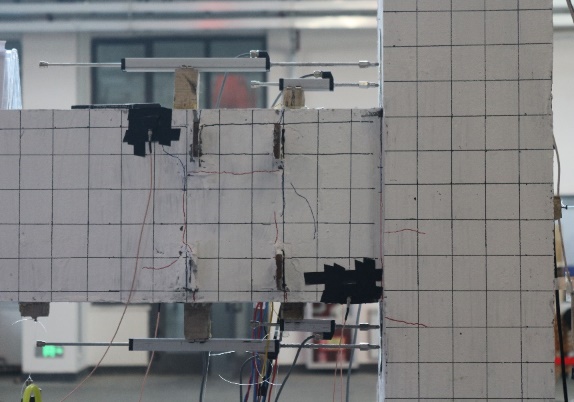 | 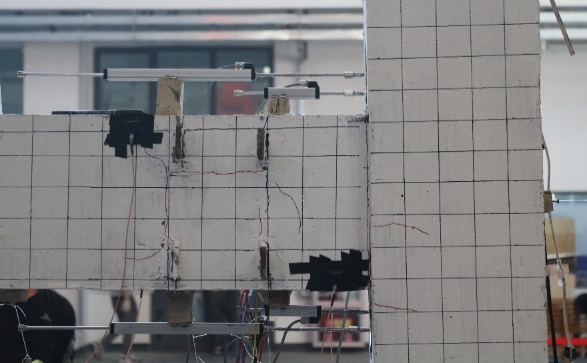 | 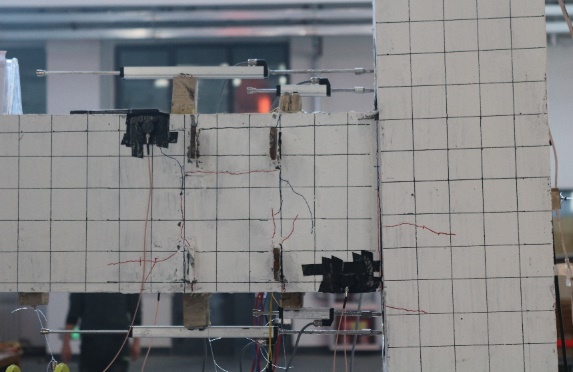 | 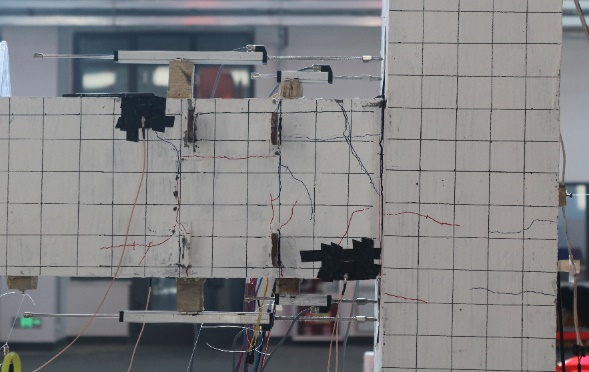 |
| --- | --- | --- | --- |
| *Δ*=1.0% | *Δ*=1.5% | *Δ*=2.0% | *Δ*=2.5% |
| 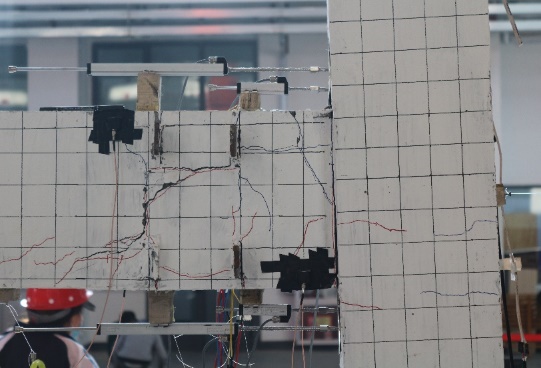 | 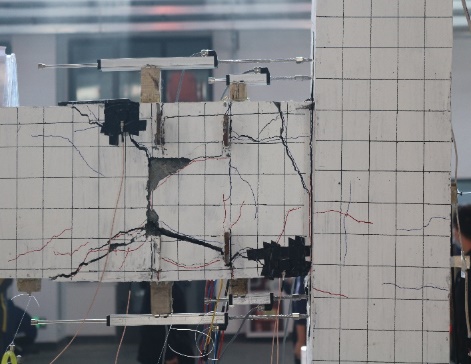 | 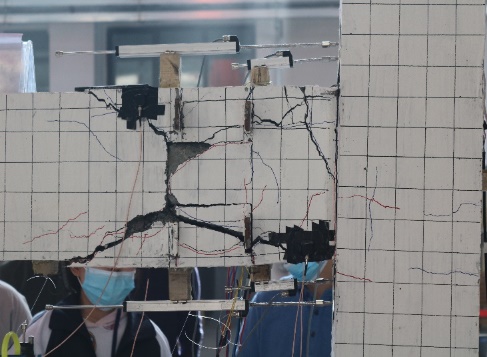 | 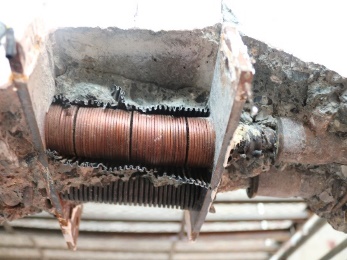 |
| *Δ*=3.0% | *Δ*=3.5% | *Δ*=4.0% | *Δ*=4.0% |

**Fig. A2.** Damage process of EPCD2.

| 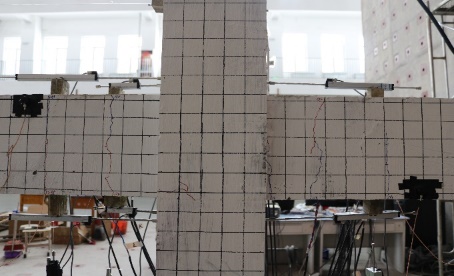 | 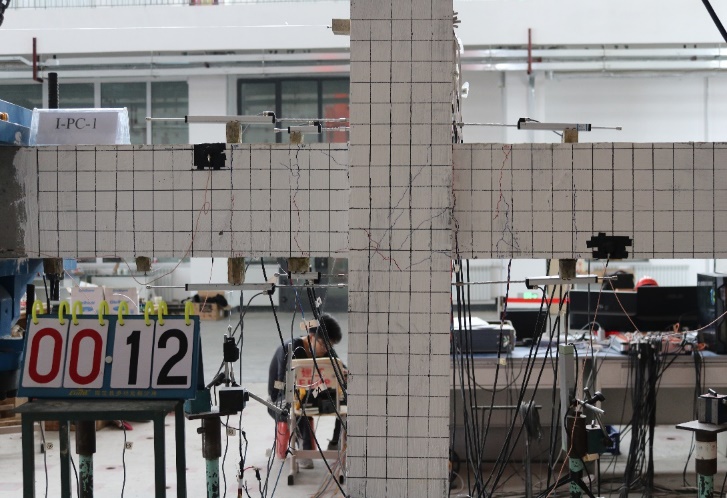 | 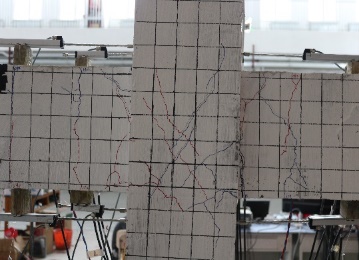 | 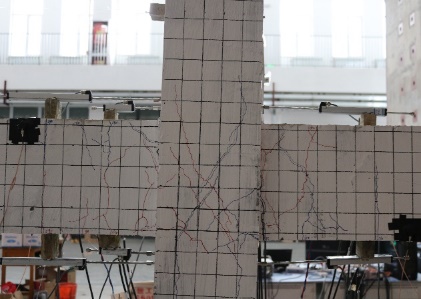 |
| --- | --- | --- | --- |
| *Δ*=1.0% | *Δ*=1.5% | *Δ*=2.0% | *Δ*=2.5% |
| 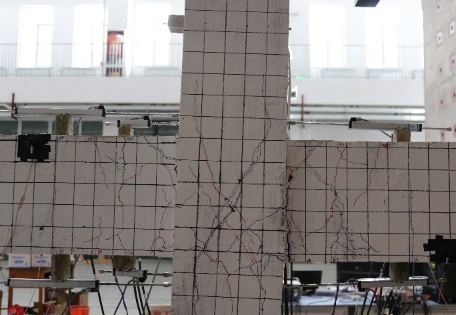 | 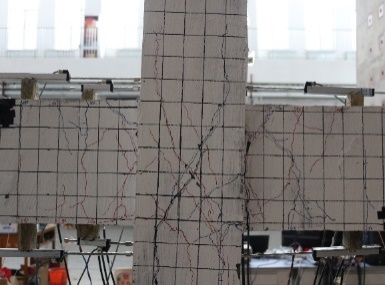 | 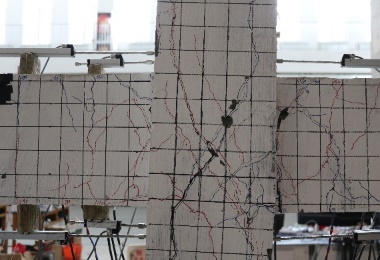 | 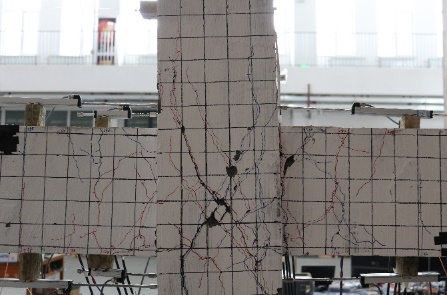 |
| *Δ*=3.0% | *Δ*=3.5% | *Δ*=4.0% | *Δ*=4.5% |
| *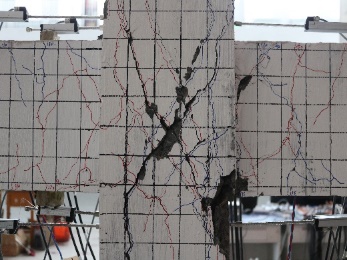* | *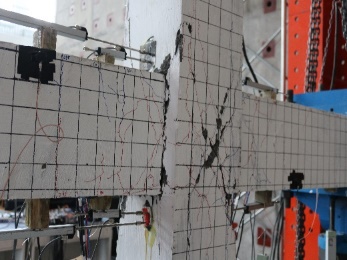* | *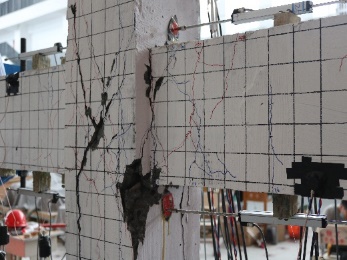* |  |
| *Δ*=5.0% | *Δ*=5.0% | *Δ*=5.0% |  |

**Fig. A3.** Damage process of IPC2.

| 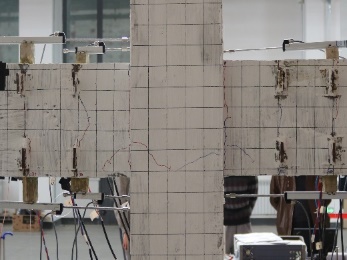 | 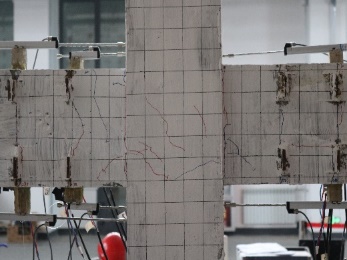 | 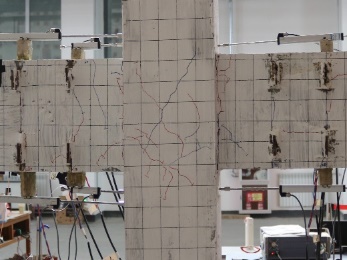 | 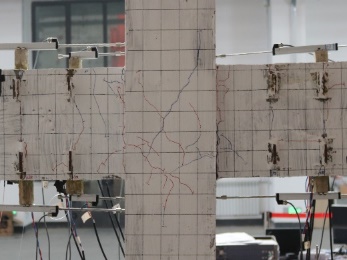 |
| --- | --- | --- | --- |
| *Δ*=1.0% | *Δ*=1.5% | *Δ*=2.0% | *Δ*=2.5% |
| 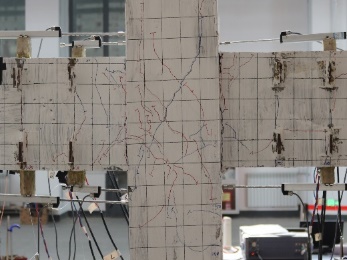 | 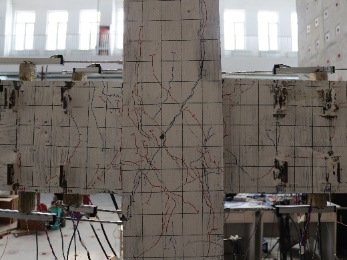 | 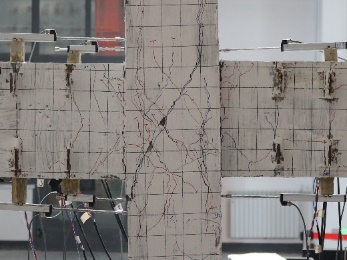 | 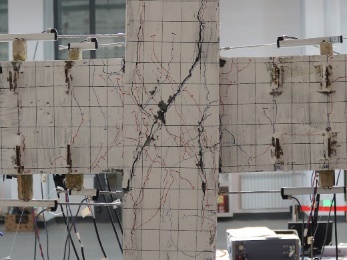 |
| *Δ*=3.0% | *Δ*=3.5% | *Δ*=4.0% | *Δ*=4.5% |
| 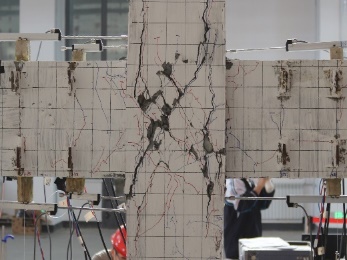 | 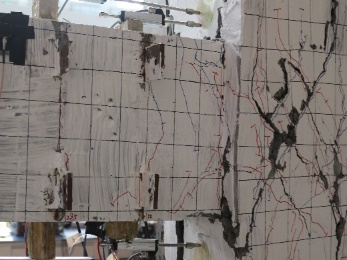 | 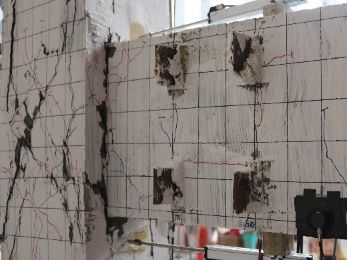 |  |
| *Δ*=5.0% | *Δ*=5.0% | *Δ*=5.0% |  |

**Fig. A4.** Damage process of IPCD2.
